# Associations of Sun Exposure with 25-Hydroxyvitamin D and Parathyroid Hormone Levels in a Cohort of Hypertensive Patients: The Graz Endocrine Causes of Hypertension (GECOH) Study

**DOI:** 10.1155/2012/732636

**Published:** 2012-02-16

**Authors:** Stefan Pilz, Katharina Kienreich, Daniel Stückler, Andreas Meinitzer, Andreas Tomaschitz

**Affiliations:** ^1^Division of Endocrinology and Metabolism, Department of Internal Medicine, Medical University of Graz, 8036 Graz, Austria; ^2^Department of Epidemiology and Biostatistics and EMGO Institute for Health and Care Research, VU University Medical Center, 1081 BT Amsterdam, The Netherlands; ^3^Clinical Institute of Medical and Chemical Laboratory Diagnostics, Medical University of Graz, 8036 Graz, Austria

## Abstract

Sunlight-induced vitamin D, synthesis in the skin is the major source of vitamin D, but data on the relationship of sun-related behaviour with vitamin D and parathyroid hormone (PTH) levels are relatively sparse. We evaluated whether habitual sun exposure is associated with 25-hydroxyvitamin D (25[OH]D) and PTH levels and whether there exist seasonal variations. We examined 111 hypertensive patients in Austria (latitude 47° N). Frequent sunbathing at home and outdoor sports were associated with higher 25(OH)D levels (*P* < 0.05 for both). Red or blond scalp hair as a child, memory of sunburns, preferring sunbathing, frequent stays on the beach or in open-air pools, and solarium use were associated with lower PTH levels (*P* < 0.05 for all). Multiple linear regression analyses including age, sex, and body mass index showed that sun exposure score was significantly associated with 25(OH)D (beta coefficient = 0.27; *P* = 0.004) and by trend with PTH (beta coefficient = −0.16; *P* = 0.09). These associations were more prominent in summer in which 25(OH)D levels were significantly higher compared to winter. Translation of these findings into recommendations for the prevention and treatment of vitamin D deficiency remains a challenge for the future.

## 1. Introduction

Vitamin D deficiency is considered a widespread public health problem because vitamin D is important for musculoskeletal health and probably also for various other chronic diseases including cardiovascular and metabolic diseases, cancer, and infections and autoimmune diseases [1–10]. A profound role of vitamin D status for human health is further supported by pathophysiological evidence showing that the vitamin D receptor (VDR) is almost ubiquitously expressed [1–10]. Furthermore, vitamin D metabolites regulate approximately three percent of the human genome [10]. The currently high worldwide prevalence of vitamin D deficiency is mainly attributable to low sunlight exposure of the skin [1–3, 11]. Previous studies suggest that ~80% of vitamin D is obtained by ultraviolet-B- (UV-B-) induced vitamin D synthesis in the skin whereas diet makes only a minor contribution [12]. Consequently, there exists a significant seasonal variation with highest 25-hydroxyvitamin D (25[OH]D) levels at the end of summer [12–15]. UV-B-induced vitamin D synthesis in the skin is, however, influenced by various factors such as skin pigmentation, age, and increasing distance from the equator or air pollution, which are all associated with reduced dermal vitamin D production [1, 12–18]. Time of day and exposed skin are also of relevance. Hence, there remain various ambiguous issues regarding the impact of habitual sunlight exposure on vitamin D status. Previous data on this topic are relatively sparse and partially inconsistent regarding the relationship of sun exposure-related behaviour and vitamin D status [19–29]. Such data are, however, clinically relevant, also in light of the fact that current recommendations for the prevention and treatment of vitamin D status mainly focus on nutritional and supplemental vitamin D leaving many physicians unsecure on how to deal with advices regarding the role of habitual sunlight exposure [2, 30, 31]. We therefore addressed the relationship of sun-related behaviour, assessed by a previously published questionnaire, with 25(OH)D levels in an Austrian cohort of hypertensive patients derived from the Graz Endocrine Causes of Hypertension (GECOH) Study [32, 33]. Our specific aims were to test (i) whether a sun exposure score is associated with 25(OH)D levels, (ii) whether this relationship is different in summer and winter, and (iii) to evaluate seasonal differences in 25(OH)D levels. In addition, we also tested whether plasma levels of parathyroid hormone (PTH), which increases in vitamin D deficiency, are associated with sun exposure, season of blood draw and 25(OH)D.

## 2. Materials and Methods

### 2.1. Study Population

The GECOH study is a diagnostic accuracy study of the aldosterone-to-active-renin ratio (AARR) in screening for primary aldosteronism (PA). The study protocol as well as baseline data of the GECOH study have been published previously [33, 34]. In this single-center study at the Medical University of Graz, Austria (latitude: 47° north), we included adult hypertensive patients (age ≥18 years), who were routinely referred to our outpatient clinic for screening for endocrine hypertension. All patients underwent standardized procedures for PA and endocrine hypertension diagnostics, as described elsewhere [33, 34]. Height and weight determinations were performed wearing light clothes and no shoes, and body mass index (BMI) was calculated according to weight (in kg) divided by the square of height (in meters). Systolic and diastolic blood pressures were measured twice by the method of Korotkoff and the mean values of both measurements were recorded. Winter season was classified from October to March and summer season was classified from April to September. We obtained written informed consent from all study participants, and the GECOH study was approved by the ethics committee at the Medical University of Graz, Austria.

### 2.2. Laboratory Methods

All laboratory procedures have been previously described in detail [33, 34]. Fasting blood samplings were performed between 8.00 and 11:00 am. Laboratory measurements were either performed on a daily or weekly basis. We measured serum 25(OH)D by a chemiluminescence assay (IDS, iSYS 25-hydroxyvitamin D; Immunodiagnostic systems Ltd., Boldon, England) on an IDS-iSYS multidiscipline automated analyser. Within-day and interday coefficients of variation (CV) were 5.5 to 12.1%, and 8.9 to 16.9%, respectively. Plasma concentrations of intact PTH were determined by electrochemiluminescence immunoassay (ECLIA) on an Elecsys 2010 (Roche Diagnostics, Mannheim, Germany), with a normal range of 15–65 pg/mL and an interassay CV of 5.7–6.3%. Serum calcium was determined by the Roche/Hitachi cobas c system analyzer (Roche Diagnostics, Mannheim, Germany). 

### 2.3. Sun Behaviour Questionnaire

We used a German language questionnaire on sun-related behaviour that was initially developed to assess the relationship of UV exposure and melanocytic nevi [32]. In detail, this evaluation (with a scoring system for the answers in parenthesis) includes the questions number 1 to 4, which concern skin reactions to sun or skin type, and the questions number 5a to 5i, which concern detailed sun-specific behaviour. Question 1: What is your personal relationship with the sun? (I avoid the sun = 0; I like the sun, but after a short time I try to protect myself or search for a shady place = 1; I love the sun and being unprotected I bear the sun for a while = 2; I could be in the sun forever = 3). Question 2: How would be your skin reaction on the first summer sun, if you would be in the midday sun for 1 hour without sun cream and without being browned by the sun before? (the next day I would have a painful sunburn and no browning after one week = 0; the next day I would have a painful sunburn and a slight browning after one week = 1; the next day I would have a slight sunburn and a moderate browning of the skin after one week = 2; the next day I would have no sunburn and a significant browning of the skin after one week = 3). Question 3: How was your scalp hair as a child? (red or blond = 0, brown or black = 1). Question 4a: How many sunburns can you describe in detail by geographical region, by body area, and by year? (no sunburn = 0; one sunburn = 1; at least two sunburns = 2). Question 4b: Did you ever have sunburns even if you cannot remember the detailed circumstances? (no = 0; yes = 1). Question 5a: Did you have frequent stays on the beach or in open-air pools? (no = 0; yes = 1). Question 5b: Did you have frequent sunbathing at home? (no = 0; yes = 1). Question 5c: Did you do regular gardening work? (no = 0, yes = 1). Question 5d: Did you do water sports or shipping? (no = 0, yes = 1). Question 5e: Did you do other outdoor sports? (no = 0; yes = 1). Question 5f: Did you do professional outdoor work? (no = 0; yes = 1). Question 5g: How many holidays did you have in sunny countries? (none = 0; one to three = 1; four to ten = 2; more than ten = 3). Question 5h: How often do you go to a solarium? (never = 0; sometimes = 1; frequently = 2). Question 5i: Have you ever been treated by light or UV therapy due to skin disease? (no = 0; yes = 1). According to the original publication of that questionnaire we calculated a “sun exposure score” as the sum of the scores of questions number 5a to 5i [32]. 

### 2.4. Statistical Analyses

Continuous data are presented as means ± standard deviation and categorical variables are presented as percentages. Student's *t*-test and analysis of variance (ANOVA) with *P* for trend was used to test for group differences in 25(OH)D or PTH levels. We performed Pearson's correlation analyses of “sun exposure score” with 25(OH)D and PTH concentrations. In addition, we performed multiple linear regression analyses with 25(OH)D levels or PTH as the dependent variable and “sun exposure score” as well as the possible confounders age (years), sex (male = 0, female = 1), and BMI (kg/m²) as independent variables. These above-described correlation and regression analyses were also performed in subgroups of patients stratified by season of blood draw. A *P* value below 0.05 was considered statistically significant and SPSS version 16.0 was used for statistical analyses.

## 3. Results

Between February 2009 and February 2011 we examined 175 Caucasian patients of the GECOH study. All study participants were asked to fill out a sun behaviour questionnaire. Questionnaires were returned by 115 patients (66% of the entire study population). Of these 115 patients we excluded 4 participants due to vitamin D supplementation, leaving 111 participants who were included into the present work. Baseline characteristics of this study cohort are presented in Table 1. Serum 25(OH)D levels were below 30 ng/mL in 53 patients (47.7%) and below 20 ng/mL in 25 patients (22.5%) (to convert 25[OH]D in ng/mL to nmol/L multiply by 2.496). In Pearson correlation analysis we observed a significant inverse association of 25(OH)D and PTH (*r* = −0.20; *P* = 0.04). Associations of 25(OH)D and PTH with answer scores of the sun behaviour questionnaire are shown in Tables 2 and 3, respectively. 25(OH)D levels were significantly increased in patients who reported on outdoor sports and sunbathing at home (see Table 2). PTH levels were significantly decreased in participants with a red or blond scalp hair as a child, in those who could recall sunburns and in those who like sunbathing and who reported on frequent stays at the beach or open-air pools and on solarium use (see Table 3). Sun exposure score was significantly associated with higher 25(OH)D levels (*r* = 0.27; *P* = 0.005) and by trend with lower PTH levels (*r* = −0.17; *P* = 0.07). In multiple linear regression analysis sun exposure score remained a significant predictor of higher 25(OH)D levels (beta coefficient = 0.27; *P* = 0.004), and this association was stronger than for BMI (beta coefficient = − 0.23; *P* = 0.01), age (beta coefficient = −0.20; *P* = 0.03), or gender (beta coefficient = 0.03; *P* = 0.73). Multiple linear regression analysis for PTH as the dependent variable showed only a nonsignificant trend for an inverse association with the sun exposure score (beta coefficient = − 0.16; *P* = 0.09). In that analysis there was no association with gender (beta coefficient = 0.06; *P* = 0.55), but age (beta coefficient = 0.34; *P* < 0.001) and BMI (beta coefficient = 0.20; *P* = 0.03) were significantly associated with PTH levels.

Serum levels of 25(OH)D (in ng/mL) were significantly higher in summer compared to winter (37 ± 18 versus 28 ± 13; *P* = 0.003) but there was no statistically significant difference in PTH levels in summer (43 ± 15 pg/mL) compared to winter (49 ± 20 pg/mL; *P* = 0.12). Season stratified analyses showed that in summer, sun exposure score was significantly associated with both 25(OH)D (*r* = 0.27; *P* = 0.03) and PTH (*r* = −0.36; *P* = 0.004). By contrast, there was no significant association in winter, neither with 25(OH)D (*r* = 0.16; *P* = 0.27) nor with PTH (*r* = 0.06; *P* = 0.67). Multiple liner regression analyses showed that sun exposure score was only by trend associated with 25(OH)D levels in summer (beta coefficient = 0.21; *P* = 0.09) and there was also no significant association in winter (beta coefficient = 0.18; *P* = 0.25). Sun exposure score, however, predicted PTH levels in summer (beta coefficient = −0.32; *P* = 0.02) but not in winter (beta coefficient = − 0.01; *P* = 0.98).

There were no significant differences in 25(OH)D levels between women (34 ± 17 ng/mL) and men (33 ± 16 ng/mL; *P* = 0.90). PTH levels were also similar in both sexes (women: 47 ± 19 pg/mL and men: 45 ± 16 pg/mL; *P* = 0.52).

## 4. Discussion

In this cohort of hypertensive patients we have shown that habitual sunlight exposure is associated with higher 25(OH)D levels and by trend with lower PTH levels. These associations were more prominent in the summer season in which 25(OH)D levels were significantly higher compared to winter.

Our work is, to the best of our knowledge, the first to address the relationship of habitual sunlight exposure with 25(OH)D and PTH levels in Central Europe. As expected, we found that higher self-reported sunlight exposure was associated with higher 25(OH)D levels, independent of the possible confounders age, sex, and BMI. These data are in line with previous studies in Southern and Northern Europe as well as other regions of the world, which also reported on a significant effect of sunlight exposure on 25(OH)D levels [19–21, 24–29]. By contrast, some investigations did not find a profound effect of sunlight-related behaviour on vitamin D status [22, 23]. Hence, although it is well established that sunlight-induced vitamin D synthesis in the skin is the major source for vitamin D, the precise impact of habitual sunlight exposure on vitamin D status remains to be further explored [15–18]. This issue is relatively complex because the efficacy of dermal vitamin D synthesis can be modulated by various factors [15–18]. Important factors are, for example, latitude and weather, which underlines the importance of our study that significantly adds to the knowledge from previous studies derived from other geographical regions. It should be acknowledged that UV-B-induced vitamin D synthesis becomes less effective with increasing distance from the equator resulting in the inability to produce vitamin D during winter season in Northern Europe. Consequently, there exists a seasonal variation of 25(OH)D that was also observed in our work and that, likewise, contributes to a significant attenuation of the association of outdoor activities and 25(OH)D in winter, as reported in a Swedish cohort [23]. This reduced dermal vitamin D synthesis in winter may underlie our observation that in season-stratified analyses of the GECOH study the correlation of sun exposure score with 25(OH)D was only significant in summer but not in winter. Higher skin pigmentation has also been reported to impair vitamin D synthesis in the skin, but we did not observe differences in 25(OH)D levels according to answers on skin type or skin reactions to the sun [15–18]. This may be due to the fact that we studied a Caucasian cohort with a relatively homogenous skin type. Regarding specific questions on sun-related behaviour, we observed that in particular outdoor sports and sunbathing at home were associated with higher 25(OH)D levels. We are well aware that this is not a very detailed assessment of sun exposure as it was done in some previous studies, but our data may provide good insight into the relationship of daily life behaviour with vitamin D status. Although our findings do not prove causality, they might be helpful for developing recommendations to change lifestyle in order to improve vitamin D status. In this context, it is important to note that previous studies have already performed detailed evaluations of the effect of artificial UV-B exposure on 25(OH)D levels [15–18, 35–38]. According to such experiments, Webb et al. calculated that when wearing knee-length shorts and a T-shirt with sleeves to mid upper arms at a latitude of 30 to 60°, midsummer noontime sunlight exposure for ~30 minutes (range 16–49 minutes) three times a week should be enough for achieving 25(OH)D levels above 20 ng/mL [35–37]. Such estimations could be a starting point for sunlight exposure advice. Current recommendations regarding sunlight exposure are, however, insufficient when considering the high prevalence of vitamin D deficiency. To address this issue Farrar et al. showed that adhering to the sunlight exposure recommendations of the UK Health Protection Agency fails to achieve a sufficient vitamin D status in UK adults of South Asian origin [38]. Apart from this, it should be taken into account that according to observational data a late summer 25(OH)D level of at least ~32 ng/mL is required to ensure 25(OH)D levels above 20 ng/mL throughout winter [36, 39]. Considering that there is an ongoing debate on the optimal 25(OH)D levels and in view of the potential adverse effects of UV exposure on diseases like skin cancer it is challenging to formulate recommendations regarding sunlight exposure for the prevention and treatment of vitamin D deficiency [35–40]. We believe that beyond giving detailed advice for minutes of sunlight exposure according to factors such as skin type, daytime, latitude, and clothing, it might also be very practical to raise more general recommendations regarding casual sunlight exposure as part of daily activities (e.g., recommending more outdoor sports activities). Concerning this, we believe that our data on habitual sunlight exposure and 25(OH)D may be helpful, and we strongly recommend further studies to evaluate the relationship of casual sunlight exposure with vitamin D status and to test whether simple advice for lifestyle changes are effective for fighting vitamin D deficiency. A previous study, however, indicates that simple advice for more sunlight exposure are by far less effective than prescription of vitamin D supplementation [41].

In addition to our findings on 25(OH)D we observed similar results for an inverse association of sun exposure and PTH levels. This result was expected due to what is known and in our study we also confirm an inverse association of 25(OH)D and PTH. Previous studies that specifically evaluated the impact of sun-related behaviour on PTH levels are, however, rare [42, 43]. Our observed inverse relationship of PTH and sun exposure was particularly pronounced in summer season with absolutely no association in winter. Specifically, we found that red or blond scalp hair as a child, memory of sunburns, and frequent stays at the beach or open-air pools were associated with low PTH levels. Interestingly, study participants who went to a solarium had also significantly decreased PTH concentrations while 25(OH)D levels were not meaningfuly different. Whereas previous investigations have already demonstrated significant effects of solaria on vitamin D status we show that there is also an association with PTH levels [44]. The clinical relevance of our PTH findings are underlined by the fact that rising PTH levels are a hallmark of vitamin D deficiency and have been associated with adverse outcomes including mortality and cardiovascular events [1–3, 44–49]. Apart from this, high PTH levels are deleterious for bone health and are also associated with calcific aortic stenosis, the metabolic syndrome, and cancer risk [50–55]. In this context, our findings provide evidence that sunlight exposure in summer might be useful for the treatment of inappropriate high PTH levels, a hypothesis that deserves further investigation. Although PTH levels are significantly and inversely correlated with 25(OH)D it should be noted that the molecular effects of PTH and vitamin D are largely independent of each other [3, 10, 56]. 

Our results are limited by the observational design of our study that limits conclusions regarding causality. Furthermore, our questionnaire on sun-related behaviour did not include evaluations for detailed calculations of UV-B exposure or some modulating and relevant factors such as sunscreen use or specific assessment of skin type. Although we excluded study participants who reported on vitamin D supplement use we did not evaluate nutritional vitamin D intake. Another possible drawback is the use of the IDS immunoassay that is not the gold standard method for the determination of 25(OH)D levels.

In conclusion, we found that habitual sun exposure in Austria is associated with higher 25(OH)D levels and, in summer, also with reduced PTH levels. These data on the relationship of habitual sun exposure with vitamin D and PTH status underline the importance of lifestyle behaviour for maintenance of physiologic vitamin D and PTH status. Translation of these findings into recommendations for changes in lifestyle to prevent and treat vitamin D deficiency remains a challenge for the near future.

## Figures and Tables

**Table 1:** Baseline characteristics of the GECOH study.

Variable	
Number	111
Age (years)	49.1 ± 15.6
Females (%)	55.0
Summer season (%)	57.7
Body mass index (kg/m²)	28.3 ± 6.0
25-hydroxyvitamin D (ng/mL)	33 ± 17
Parathyroid hormone (pg/mL)	46 ± 17
Serum calcium (mmol/L)	2.34 ± 0.09
Systolic blood pressure (mm Hg)	153 ± 24
Diastolic blood pressure (mm Hg)	95 ± 14
Primary aldosteronism (%)	3.6

Continuous data are presented as means ± standard deviation, and categorical data are presented as percentages.

**Table 2:** Associations of 25-hydroxyvitamin D with answers from a sun behaviour questionnaire.

Question number	Question content	Answer scores				*P* value
		0	1	2	3	
1	Personal sun relationship	28 ± 6	31 ± 15	37 ± 19	38 ± 23	0.06
		*n* = 8	*n* = 60	*n* = 34	*n* = 9	
2	Skin reaction to sun	28 ± 12	33 ± 16	35 ± 16	33 ± 20	0.25
		*n* = 17	*n* = 25	*n* = 49	*n* = 20	
3	Scalp hair as a child	33 ± 15	34 ± 17			0.79
		*n* = 42	*n* = 69			
4a	Memory of sunburns	33 ± 17	34 ± 16	34 ± 18		0.68
		*n* = 51	*n* = 39	*n* = 21		
4b	Ever sunburns	18 ± 8	34 ± 17			0.07
		*n* = 4	*n* = 107			
5a	Beach or open-air pools	31 ± 14	37 ± 19			0.05
		*n* = 63	*n* = 48			
5b	Sunbathing at home	31 ± 14	38 ± 21			0.04
		*n* = 73	*n* = 38			
5c	Gardening	35 ± 17	32 ± 16			0.37
		*n* = 68	*n* = 43			
5d	Water sports or shipping	34 ± 17	31 ± 13			0.55
		*n* = 99	*n* = 12			
5e	Other outdoor sports	30 ± 15	40 ± 18			0.003
		*n* = 74	*n* = 37			
5f	Outdoor work	32 ± 16	36 ± 18			0.29
		*n* = 85	*n* = 26			
5g	Holidays in sunny countries	29 ± 15	30 ± 16	34 ± 16	36 ± 17	0.07
		*n* = 13	*n* = 23	*n* = 21	*n* = 54	
5h	Solarium	33 ± 17	34 ± 16			0.92
		*n* = 92	*n* = 19	*n* = 0		
5i	Light or UV therapy	33 ± 16	35 ± 24			0.87
		*n* = 106	*n* = 5			

25-hydroxyvitamin D data are shown in ng/mL as means ± standard deviation.

*P* values are derived from either Student's *t*-test or from analysis of variance (ANOVA) with *P* for trend.

**Table 3:** Associations of parathyroid hormone with answers from a sun behaviour questionnaire.

Question number	Question content	Answer scores				*P* value
		0	1	2	3	
1	Personal sun relationship	50 ± 17	49 ± 19	41 ± 14	42 ± 19	0.04
		*n* = 8	*n* = 60	*n* = 34	*n* = 9	
2	Skin reaction to sun	41 ± 16	50 ± 16	45 ± 18	47 ± 19	0.71
		*n* = 17	*n* = 25	*n* = 49	*n* = 20	
3	Scalp hair as a child	41 ± 14	49 ± 19			0.03
		*n* = 42	*n* = 69			
4a	Memory of sunburns	50 ± 19	42 ± 17	43 ± 14		0.048
		*n* = 51	*n* = 39	*n* = 21		
4b	Ever sunburns	53 ± 32	45 ± 17			0.38
		*n* = 4	*n* = 107			
5a	Beach or open air pools	50 ± 16	40 ± 17			0.003
		*n* = 63	*n* = 48			
5b	Sunbathing at home	46 ± 18	46 ± 16			0.97
		*n* = 73	*n* = 38			
5c	Gardening	46 ± 17	46 ± 18			0.88
		*n* = 68	*n* = 43			
5d	Water sports or shipping	46 ± 18	44 ± 11			0.60
		*n* = 99	*n* = 12			
5e	Other outdoor sports	46 ± 17	45 ± 18			0.71
		*n* = 74	*n* = 37			
5f	Outdoor work	44 ± 17	50 ± 19			0.15
		*n* = 85	*n* = 26			
5g	Holidays in sunny countries	51 ± 16	43 ± 19	52 ± 21	43 ± 15	0.28
		*n* = 13	*n* = 23	*n* = 21	*n* = 54	
5h	Solarium	48 ± 18	36 ± 12			0.006
		*n* = 92	*n* = 19	*n* = 0		
5i	Light or UV therapy	46 ± 18	41 ± 12			0.58
		*n* = 106	*n* = 5			

Parathyroid hormone data are shown in ng/mL as means ± standard deviation.

*P* values are derived from either Student's *t*-test or from analysis of variance (ANOVA) with *P* for trend.
